# A Thia-Analogous Indirubin *N*-Glycoside Disrupts Mitochondrial Function and Causes the Death of Human Melanoma and Cutaneous Squamous Cell Carcinoma Cells

**DOI:** 10.3390/cells12192409

**Published:** 2023-10-05

**Authors:** Franziska Wendt, Felix Wittig, Anne Rupprecht, Robert Ramer, Peter Langer, Steffen Emmert, Marcus Frank, Burkhard Hinz

**Affiliations:** 1 Institute of Pharmacology and Toxicology, Rostock University Medical Centre, 18057 Rostock, Germany; franziska.wendt@med.uni-rostock.de (F.W.); felix.wittig@med.uni-rostock.de (F.W.); rupprechta31@gmail.com (A.R.); robert.ramer@med.uni-rostock.de (R.R.); 2 Institute of Organic Chemistry, University of Rostock, 18059 Rostock, Germany; peter.langer@uni-rostock.de; 3 Clinic and Policlinic for Dermatology, Rostock University Medical Centre, 18057 Rostock, Germany; steffen.emmert@med.uni-rostock.de; 4 Electron Microscopy Centre, Rostock University Medical Centre, 18057 Rostock, Germany; marcus.frank@med.uni-rostock.de; 5 Department Life, Light and Matter, University of Rostock, 18059 Rostock, Germany

**Keywords:** thia-analogous indirubin *N*-glycoside, skin cancer, melanoma cells, squamous cell carcinoma cells, heme oxygenase-1, mitochondria, oxygen consumption rate, respiratory chain complex proteins

## Abstract

Skin cancer is the most common malignant disease worldwide and, therefore, also poses a challenge from a pharmacotherapeutic perspective. Derivatives of indirubin are an interesting option in this context. In the present study, the effects of 3-[3′-oxo-benzo[*b*]thiophen-2′-(*Z*)-ylidene]-1-(β-d-glucopyranosyl)-oxindole (KD87), a thia-analogous indirubin *N*-glycoside, on the viability and mitochondrial properties of melanoma (A375) and squamous cell carcinoma cells (A431) of the skin were investigated. In both cell lines, KD87 caused decreased viability, the activation of caspases-3 and -7, and the inhibition of colony formation. At the mitochondrial level, a concentration-dependent decrease in both the basal and ATP-linked oxygen consumption rate and in the reserve capacity of oxidative respiration were registered in the presence of KD87. These changes were accompanied by morphological alterations in the mitochondria, a release of mitochondrial cytochrome c into the cytosol and significant reductions in succinate dehydrogenase complex subunit B (SDHB, subunit of complex II) in A375 and A431 cells and NADH:ubiquinone oxidoreductase subunit B8 (NDUFB8, subunit of complex I) in A375 cells. The effect of KD87 was accompanied by a significant upregulation of the enzyme heme oxygenase-1, whose inhibition led to a partial but significant reduction in the metabolic-activity-reducing effect of KD87. In summary, our data show a mitochondria-targeting effect of KD87 as part of the cytotoxic effect of this compound on skin cancer cells, which should be considered in future studies with this class of compounds.

## 1. Introduction

Skin cancer is by far the most common of all cancers. It includes basal cell carcinoma (BCC) and squamous cell carcinoma (SCC), accounting for about 77% (BCC) and 20% (SCC) of all cases, and melanoma, which is responsible for most skin cancer-related deaths, although it accounts for only 1% of all skin cancer cases [1,2]. While the surgical removal of tumours in patients diagnosed with primary melanoma is the best chance of a definitive cure, late-stage metastatic melanoma remains a major challenge. In the last decade, success has been achieved with chemotherapy, targeted therapy and immunotherapy (for review see [1]). Nevertheless, although the mortality rate for melanoma decreased by 5.7% annually between 2014 and 2018 [3], the most recent Global Cancer Observatory (GLOBOCAN) report listed 324,635 new cases of melanoma of the skin with 57,043 deaths in 2020 [4]. For non-melanoma skin cancer excluding cases of BCC, GLOBOCAN reported 1,198,073 cases with 63,731 deaths. These data clearly show the need to develop new therapeutic options in this field (for review see [2,5]).

From this perspective, indirubin derivatives represent an interesting option for use in skin cancer (for review see [2]). The red-coloured indirubin, a 3,2′ bisindole isomer of indigo (blue dye, 2,2′-bisindol), has been identified as an active ingredient in Danggui Longhui Wan, a mixture of plants used in traditional Chinese medicine, and has shown significant anti-cancer activity in clinical trials with patients suffering from chronic myelocytic leukaemia (CML) and chronic granulocytic leukaemia ([6], for review see [7,8]). In a phase III clinical trial with CML patients, the oral administration of meisoindigo, a later synthesised better-tolerated indirubin derivative, resulted in haematological complete and partial response rates of 45.0 and 39.3%, respectively, in newly diagnosed patients, and 35.9 and 41.4%, respectively, in previously treated patients [9]. Furthermore, according to a retrospective analysis, meisoindigo shows a synergistic effect with hydroxyurea in CML patients [10].

Meanwhile, a number of intracellular indirubin targets such as cyclin-dependent kinases [11,12], glycogen synthase kinase 3 [13] or signal transducer and activator of transcription-3 [14,15] have been identified, in particular for indirubin-3′-monoxime (I3M), which could mediate corresponding antitumorigenic effects. Interest has also been aroused by *N*-glycosides of indirubin, first isolated by the group of Laatsch from terrestrial *Streptomyces* sp. GW 48/1497 [16]. Thereby, subsequently synthesised indirubin *N*-glycosides [17], thia-analogous indirubin *N*-glycosides [18,19,20,21] and *N*-glycosylated 3-alkylideneoxindoles [22,23,24] exhibited antiproliferative and cytotoxic activity on cells of various tumour entities, with melanoma cells also having been successfully investigated in this regard for the latter two chemical substance groups.

Regarding the impact of indirubins on tumour cells, the induction of apoptosis has been repeatedly pointed out, with several studies demonstrating the involvement of mitochondrial processes in indirubin-induced tumour cell death [15,19,24,25,26]. However, possible causes of mitochondrial dysfunction with regard to the mitochondrial respiratory chain have not yet been investigated. In the present paper, the effect of KD87 (3-[3′-oxo-benzo[*b*]thiophen-2′-(*Z*)-ylidene]-1-(β-d-glucopyranosyl)-oxindole), a thia-analogous indirubin *N*-glycoside, was comparatively investigated with regard to its cytotoxic and mitochondria-targeting effect in human melanoma cells (A375) and human cutaneous SCC cells (A431). Here, for the first time for an indirubin derivative, we were able to show a decrease in basal and ATP-linked oxygen consumption rates as well as in the reserve capacity of oxidative respiration, which was accompanied by a reduction in certain subunits of respiratory complexes. These results provide evidence for previously unknown targets of an indirubin derivative.

## 2. Materials and Methods

### 2.1. Materials

The thia-analogous indirubins KD87 and KD88 were synthesised by the group of Prof. Peter Langer (Institute of Organic Chemistry, University of Rostock, Rostock, Germany). Leupeptin was obtained from Biomol (Hamburg, Germany). Tin protoporphyrin IX dichloride (SnPPIX; #ALX-430-051-M005) and copper(II) protoporphyrin IX (CuPPIX; #ALX-430-049-M025) were purchased from Enzo Life Sciences (Lörrach, Germany). Aprotinin, bromophenol blue, hydrogen peroxide solution (H_2_O_2_, 30%), luminol, *N*-acetyl-L-cysteine (NAC) (#A7250-5G), orthovanadate, *p*-coumaric acid, phenylmethanesulfonyl fluoride (PMSF) and penicillin–streptomycin were obtained from Sigma-Aldrich (Taufkirchen, Germany). Aqua ad iniectabilia was bought from Braun Melsungen (Melsungen, Germany). Methanol was acquired from J. T. Baker (Griesheim, Germany). Acetic acid, dimethyl sulfoxide (DMSO), ethylenediaminetetraacetic acid (EDTA), glycerin, glycine, hydrochloric acid 37% (HCl), Ponceau S, sodium chloride (NaCl), sodium hydroxide (NaOH), sodium dodecyl sulphate (SDS) ultra-pure, Tris ultrapure and Tris hydrochloride (Tris HCl) were purchased from AppliChem (Darmstadt, Germany). The compounds 4-(2-hydroxyethyl)-1-piperazineethanesulfonic acid (HEPES) and β-mercaptoethanol were obtained from Ferak Berlin (Berlin, Germany). Acrylamide (Rotiphorese^®^ Gel, 30%), albumin (IgG-free), ammonium peroxydisulphate (APS), crystal violet, *N*,*N*,*N*′,*N*′-tetramethylethylenediamine (TEMED), Triton^®^ X-100 and Tween^®^ 20 were obtained from Carl Roth (Karlsruhe, Germany). Non-fat milk (NFM) powder was bought from Bio-Rad Laboratories (Munich, Germany). High-glucose Gibco™ DMEM (4.5 g/L glucose, GlutaMAX™-I supplement and pyruvate; #31966021) was purchased from ThermoFisher Scientific (Schwerte, Germany). Dulbecco’s phosphate-buffered saline (DPBS) was purchased from PAN-Biotech (Aidenbach, Germany). Fetal bovine serum (FBS) superior was purchased from Bio&Sell (Feucht by Nürnberg, Germany).

### 2.2. Cell Culture

The human melanoma cell line A375 (#300110; RRID:CVCL_0132), the human skin epidermoid carcinoma cell line A431 (#300112; RRID:CVCL_0037) and the non-tumorigenic immortilised keratinocyte cell line HaCaT (#300493; RRID:CVCL_0038) were purchased from CLS Cell Lines Service (Eppelheim, Germany).

All cell lines were cultured in DMEM (high glucose, GlutaMAX^TM^-I supplement, pyruvate) supplemented with 10% (*v*/*v*) heat-inactivated FBS superior, 100 U/mL penicillin, and 100 μg/mL streptomycin (hereafter referred to as serum-containing DMEM) in a humidified incubator at 37 °C and 5% CO_2_. Incubation with specific compounds was carried out in serum-free DMEM supplemented with 100 U/mL penicillin and 100 μg/mL streptomycin (hereafter referred to as serum-free DMEM) and was performed after washing the cells with DPBS. The only exception was the colony formation assays, where the incubation with test substances was performed in serum-containing DMEM.

The test substances were dissolved in DMSO (KD87, KD88, CuPPIX), DPBS (NAC) or 1 M NaOH (SnPPIX). The final concentration of solvents in the incubation media of cells treated with test substances and vehicle did not exceed 1% (*v*/*v*) for DMSO and 0.4 mM for NaOH. In all experiments, the incubation media of vehicle- and substance-treated cells contained the same amount of solvent.

### 2.3. Metabolic-Activity and Cell-Death Assay

For viability analyses, A375, A431 or HaCaT cells were seeded in 96-well plates at a density of 5000 cells per well in serum-containing DMEM and cultured for 24 h. The cells were then treated with the test substances for 24 h in serum-free DMEM.

The metabolic activity of A375 and A431 cells was determined using the water-soluble tetrazolium salt WST-1 (4-[3-(4-iodophenyl)-2-(4-nitrophenyl)-2H-5-tetrazolio]-1,3-benzene disulfonate, Roche Diagnostics, Mannheim, Germany). This assay is based on the cleavage of WST-1 to a soluble formazan dye, whereby this bioreduction is largely dependent on the production of NAD(P)H in viable cells. Here, the amount of formazan dye formed correlates directly with the number of metabolically active cells in the culture. WST-1 was added at a final dilution of 1:10. The cells were then incubated further for 20 min before the absorbance at 450 nm (wavelength correction at 690 nm) was measured with a microplate reader (Infinite F200 Pro Tecan, Tecan Group, Männedorf, Switzerland).

Cell death was indirectly quantified with the crystal violet assay. This assay is based on the property of crystal violet, an intercalating dye, to bind to cellular proteins and DNA. The crystal violet staining decreases with the death of the cells, which lose their adherence and detach from the cell culture plates accordingly. In this way, the method can be used to determine the number of remaining viable adherent cells. To this end, A375, A431 or HaCaT cells were fixed overnight with ice-cold absolute ethanol before incubation with crystal violet staining solution (0.1% [*w*/*v*] crystal violet in 10% ethanol) for 30 min. After excess dye was thoroughly washed off, the stained cells were dissolved with 10% acetic acid and the dye intensity was measured at 570 nm using a microplate reader.

### 2.4. Caspase Assay

Analysis of caspase-3 and -7 activity was performed using Caspase-Glo^®^ 3/7 Assay (#G8093; Promega). For this purpose, A375, A431 or HaCaT cells were seeded in 96-well plates at a density of 5000 cells per well and cultured in serum-containing DMEM for 24 h. The cells were then incubated with the appropriate test substance for the indicated time in serum-free DMEM. Afterwards, the Caspase-Glo^®^ 3/7 reagent was added to the wells according to the manufacturer’s instructions, followed by a 1 h incubation in the dark at room temperature. Luminescence was measured with a microplate reader.

### 2.5. Colony Formation Assay

To demonstrate colony-forming properties, 500 cells (A375 or A431) per well of a 6-well plate were seeded in serum-containing DMEM. After 24 h, treatment with vehicle or the appropriate concentrations of KD87 or KD88 was performed. After an incubation period of 7 days in serum-containing DMEM, cells were fixed in ethanol, stained with 0.1% (*w*/*v*) crystal violet and images were taken with a camera. The colonies were counted as a total number.

### 2.6. Cell Invasion and Migration

Cell invasiveness was quantified using a modified Boyden chamber assay, as described before [27] with slight modifications, and Falcon^®^ cell culture inserts (8 µm pore size; Corning, Corning, NY, USA). In this assay, cells seeded into the inserts must pass through a Matrigel layer by proteolytic degradation and then migrate through a polyethylene terephthalate membrane with 8 µm pores to a chemoattractant (DMEM 10% [*v*/*v*] FBS) in the 24-well companion plate (lower compartment). In brief, the upper sides of the inserts were coated with 70 µL (corresponds to 26.32 µg Matrigel) of a Matrigel–DPBS solution (Corning^®^ Matrigel^®^ Matrix (BD Biosciences, Heidelberg, Germany; #356234)) per insert and prepared 24 h prior to the incubation of A375 cells. To start the experiment, cells were placed at a density of 250,000 cells in a final volume of 500 µL serum-free DMEM per insert. Cells were immediately treated with KD87 or vehicle and were incubated for 72 h. After incubation, the non-migrated cells remaining on the top of the inserts were removed along with the Matrigel layer with a cotton swab. Migrated cells adhering to the bottom of the insert were quantified using the WST-1 colorimetric assay. Migration assays were carried out in parallel in the same manner, but with uncoated inserts. Viability tests, which were performed as WST-1 assays after 72 h of incubation in context of the invasion and migration analyses, were used to exclude nonspecific toxic effects under similar conditions of a high cell density (250,000 cells in a final volume of 500 µL of serum-free DMEM per well of a 48-well plate).

### 2.7. Seahorse XFe Analysis

The oxygen consumption rate (OCR) and extracellular acidification rate (ECAR) were determined using the Seahorse XFe24 analyser (Agilent Technologies, Waldbronn, Germany) according to the manufacturer’s instructions. For this purpose, A375 and A431 cells were seeded on Seahorse 24XFe plates (Agilent Technologies) at a density of 25,000 cells per well and incubated in serum-containing DMEM for 24 h. After washing with DPBS, the cells were treated with KD87 or vehicle for 24 h in serum-free DMEM. At the start of Seahorse XFe analysis, the medium was changed to unbuffered XF base medium, pH 7.4 (#103575-100, Agilent Technologies) supplemented with 10 mM glucose, 2 mM glutamine and 1 mM pyruvate. Unbuffered XF base medium remained on the cells for another 1 h. According to the manufacturer’s instructions and own cell line-specific preliminary experiments, compounds from the Seahorse XF Cell Mito Stress Test Kit (#133015-100, Agilent Technologies) were then added to the wells (port A: 1.5 μM oligomycin (ATP synthase inhibitor); port B: 2.5 μM (for A375 cells) or 1 µM (for A431 cells) FCCP (carbonyl cyanide-4 (trifluoromethoxy) phenylhydrazone; uncoupling agent that collapses the proton gradient and disrupts the mitochondrial membrane potential); port C: 0.5 μM rotenone (complex I inhibitor) and 0.5 μM antimycin A (complex III inhibitor)). OCR and ECAR per well were normalised to the respective protein amount determined after the experiment. To this end, 10 µL of lysis buffer (50 mM HEPES, 150 mM NaCl, 1 mM EDTA, 1% [*v*/*v*] Triton^®^ X-100, 10% [*v*/*v*] glycerol, 10 µg/mL aprotinin, 1 µg/mL leupeptin, 1 mM orthovanadate and 1 mM PMSF) was added to each well and the lysates were collected. Protein concentration was determined using the Pierce™ BCA Protein Assay Kit (Thermo Fisher Scientific).

Basal respiration, ATP production-based respiration, spare capacity and proton leak were calculated using the Seahorse XF Cell Mito Stress Test Report Generator (Agilent Technologies).

### 2.8. Total Cellular Protein Isolation

A375 or A431 were seeded in 6-well plates at a density of 200,000 cells per well and cultured for 24 h in serum-containing DMEM before treatment with compounds in serum-free DMEM. After completion of each cell incubation, the supernatants were collected, the cells were washed with DPBS and detached with trypsin-EDTA. Supernatant, DPBS wash and detached cells were pelleted using centrifugation (200× *g*, 4 °C) for 5 min. The cell pellet was then washed with DPBS and centrifuged again for 5 min (250× *g*, 4 °C). Afterwards, the cell pellet was mixed with lysis buffer (50 mM HEPES, 150 mM NaCl, 1 mM EDTA, 1% [*v*/*v*] Triton^®^ X-100, 10% [*v*/*v*] glycerol, 10 µg/mL aprotinin, 1 µg/mL leupeptin, 1 mM orthovanadate, 1 mM PMSF), incubated overnight at −20 °C and then centrifuged (20,817× *g*, 4 °C) for 5 min. The resulting supernatant, containing total cellular protein, was collected and stored for further protein analysis. Protein concentration was measured using the Pierce™ BCA Protein Assay Kit.

### 2.9. Mitochondrial Protein Isolation

A375 or A431 cells were seeded, treated and harvested as in the whole-cell protein isolation experiments. The resulting cell pellet was washed in 0.9% NaCl (5 min, 500× *g*, 4 °C). The Qproteome Mitochondria Isolation Kit (Qiagen, Hilden, Germany) was used to isolate mitochondria and obtain the cytosolic protein fraction according to the manufacturer’s instructions for standard preparation. To extract the mitochondrial protein, the resulting mitochondrial fraction was treated with lysis buffer, centrifuged and collected in a similar manner to the protocol used to isolate total cellular protein.

### 2.10. Western Blot Analysis

Equal amounts of protein were separated on an 8% (PARP analysis) or 12% (all other proteins) SDS–polyacrylamide gel, transferred to a nitrocellulose membrane and incubated for 1 h in 5% (*w*/*v*) NFM in Tris-buffered saline containing 0.1% (*v*/*v*) Tween^®^ 20 (TBS-T buffer). Following washing with TBS-T buffer, membranes were incubated overnight at 4 °C with primary antibodies in 1% (*w*/*v*) NFM. The OxPhos Human WB Antibody Cocktail (#45-8199) was purchased from Thermo Fisher Scientific. Other antibodies used were against PARP (#9532) and Cytochrome c (#11940), both from Cell Signaling Technology (Frankfurt/Main, Germany), VDAC1/Porin + VDAC3 (#ab14734) from Abcam (Berlin, Germany), HO-1 (#ADI-SPA-894-F) and HO-2 (#ADI-SPA-897-F), both from Enzo Life Sciences, and GAPDH (#G9545) and β-Actin (#A5441), both from Sigma-Aldrich. After washing with TBS-T buffer, membranes were incubated with secondary antibodies coupled to horseradish peroxidase (anti-rabbit antibody, #7074; anti-mouse antibody, #7076; Cell Signaling Technology) in 1% (*w*/*v*) NFM in TBS-T buffer for 1 h at room temperature. For visualisation of antibody binding, a chemiluminescent solution (100 mM Tris hydrochloride pH 8.5, 1.25 mM luminol, 200 µM *p*-coumaric acid, 0.09% [*v*/*v*] H_2_O_2_) was added and signal detection was performed using the ChemiDoc XRS gel documentation system (Bio-Rad Laboratories, Munich, Germany). Quantification of signal intensity was performed using the Quantity One 1-D Analysis Software Version 4.6.6 (Bio-Rad Laboratories). The signal of a specific protein band was normalised to the signal of the loading control. Accordingly, the protein levels of VDAC for mitochondrial proteins, GAPDH for cytosolic proteins or β-actin for total cellular protein were also analysed. Protein levels were then calculated relative to the vehicle control. The Precision Plus Protein™ Dual Colour Standard (Bio-Rad Laboratories) was used to determine the molecular weight of the bands. Upon completion of the analysis, the membranes were stripped and reprobed.

### 2.11. Quantitative Reverse Transcriptase Polymerase Chain Reaction (RT-PCR)

For quantification of mRNA expression of HO-1 and HO-2, A375 or A431 were seeded, treated and harvested as in the whole cell protein isolation experiments. Total RNA was isolated using the RNeasy Mini Kit from Qiagen (Hilden, Germany). The mRNA levels of HO-1, HO-2 and peptidylprolyl isomerase A (PPIA) were determined using real-time RT-PCR using the Applied Biosystems^®^ TaqMan^®^ RNA-to-CT™ 1-Step Kit (Thermo Fisher Scientific). Primers and probes for human PPIA (Assay ID: Hs999904_m1; VIC-MGB), HO-1 (Assay ID: Hs01110251_m1; FAM-MGB) and HO-2 (Assay ID: Hs00157969_m1; FAM-MGB) were Applied Biosystems^®^ TaqMan^®^ Gene Expression Assay products (Thermo Fisher Scientific). All experiments were performed according to the manufacturer’s instructions. As a housekeeping gene, PPIA was used to normalise the mRNA levels of HO-1 and HO-2 prior to comparison with the corresponding vehicle controls.

### 2.12. Electron Microscopy

A375 or A431 cells were seeded at a density of 200,000 cells per well in a 6-well-plate, cultured serum-containing DMEM for 24 h. Thereafter, the cells were incubated with KD87, KD88 or vehicle for 24 h in serum-free DMEM and then treated as described for the protein extraction experiments. Cell pellets were fixed in a solution containing 2% glutaraldehyde and 1% paraformaldehyde in 0.1 M phosphate buffer pH 7.3 and stored at 4 °C until further processing for embedding. After washing twice in 0.1 M phosphate buffer, cells were mixed with prewarmed 2% low melting agarose (Sigma-Aldrich) in 0.05 M HEPES buffer at 40 °C, collected by centrifugation and, after hardening, enclosed as a pellet in the agarose. The specimen blocks were then post-fixed in a 1% osmium tetroxide solution (Carl Roth) for 2 h and after washing in distilled water dehydrated in an ascending series of acetone. Resin infiltration started with overnight incubation in a 1:1 mixture of acetone and Epon resin (48% Epon 812, 30% methylnadic anhydride, 20.7% 2-dodecenylsuccinic acid anhydride, 1.3% 2,4,6-tris(dimethylaminomethyl)phenol; all components were from Serva, Heidelberg, Germany), followed by pure Epon resin for 4 h. Specimen blocks were transferred to silicone rubber moulds with fresh resin and cured in an oven at 60 °C for 2 days. Further processing included the trimming of the resin blocks (Leica EM Trim2, Leica Microsystems, Wetzlar, Germany) and subsequent sectioning on an ultramicrotome (Ultracut S, Reichert, Wien, Austria) using a diamond knife (Diatome, Nidau, Switzerland). To visualise and select the areas for ultrastructural examination, semithin sections with a thickness of 0.5 µm were stained with toluidine blue. Ultrathin sections of about 50–70 nm thickness were transferred to copper grids and stained with uranyl acetate and lead citrate before being examined with a Zeiss EM902 transmission electron microscope operated at 80 kV (Carl Zeiss, Oberkochen, Germany). Digital images were acquired with a side-mounted 1x2k FT-CCD Camera (Proscan, Scheuring, Germany) using iTEM camera control and imaging software (iTEM version number 1187, Olympus Soft Imaging Solutions, Münster, Germany). The quantification of the damaged mitochondria was performed visually using the electron microscopy images. For this purpose, the integrity of the mitochondrial membrane, the degree of swelling and the integrity of the cristae structure were examined. The percentage of damaged mitochondria relative to the total number of mitochondria analysed is shown.

### 2.13. Statistics

All statistical analyses were performed using GraphPad Prism 9.3.0 (GraphPad Software, San Diego, CA, USA). Comparisons between more than two groups were conducted using one-way ANOVA with Dunnett’s post hoc test, when all conditions were compared with a vehicle control or using Bonferroni’s post hoc test for selected group comparisons. For a simplified presentation, the determination of statistical significance was limited to the groups of interest.

## 3. Results

### 3.1. The Thia-Analogous Indirubin N-Glycoside Derivative KD87, but Not Its Non-Glycosylated Structural Analogue KD88, Causes the Concentration-Dependent Death of Human Melanoma (A375) and Human Cutaneous Squamous Carcinoma Cells (A431)

**Figure 1:**
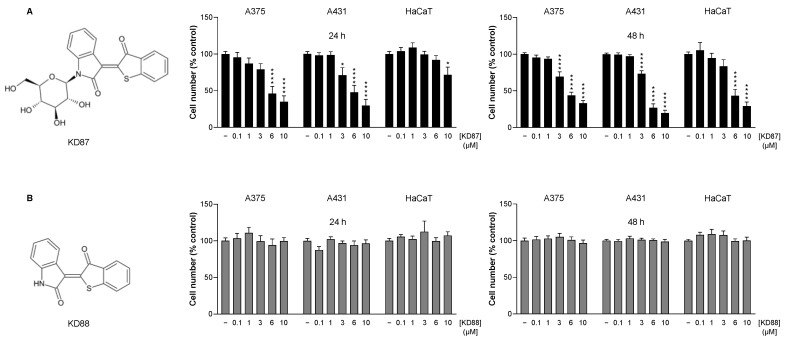
Effect of KD87 (**A**) and KD88 (**B**) on the viability of A375, A431 and HaCaT cells. Cells were incubated with KD87 or KD88 at the indicated concentrations for 24 h (**left**) or 48 h (**right**). The values given in the diagrams are based on the crystal violet staining of viable cells. All percentage values shown refer to the respective vehicle control, which was set to 100%. The data are mean values ± SEM of *n* = 9 per group from 3 independent experiments ((**A**), left, HaCaT; (**B**), left and right, HaCaT), *n* = 12 per group from 4 independent experiments ((**A**), left, A375 and A431; (**A**), right, HaCaT; (**B**), left, A375 and A431), *n* = 18 per group from 6 independent experiments ((**B**), right, A375 and A431) or *n* = 21 from 7 independent experiments ((**A**), right, A375 and A431). * *p* ≤ 0.05, **** *p* ≤ 0.0001 vs. corresponding vehicle control; one-way ANOVA with Dunnett’s post hoc test.

As we have recently shown, the *N*-glycosylated thia-analogous indirubin KD87, but not its non-glycosylated derivative KD88 (structural formulas in Figure 1), exerts a concentration-dependent inhibitory effect on the metabolic activity of A375 and A431 cells in the WST-1 assay [20]. To confirm the differential effects of KD87 and KD88 on viability, a corresponding response was again examined using the crystal violet staining of viable cells after a 24 and 48 h incubation of A375 melanoma cells and A431 squamous carcinoma cells with the respective compounds. HaCaT keratinocytes were used as a non-malignant control cell line.

As shown in Figure 1A,B, there was a concentration-dependent cytotoxic effect for KD87, but not for KD88, in A375 and A431 cells, which could be detected after 24 and 48 h of treatment. In HaCaT keratinocytes, no cytotoxicity was detected at least up to a KD87 concentration of 6 µM after 24 h of incubation (Figure 1A, left). However, after 48 h of treatment, a significant decrease in cell number was observed in HaCaT cells when incubated with 6 and 10 µM KD87, which was slightly less pronounced than when A431 cells were treated (Figure 1A, right). No significant toxicity was observed for HaCaT in the presence of 3 µM KD87, although this concentration induced significant toxicity in A375 and A431 cells (Figure 1A, right). As with A375 and A431 cells, KD88 also showed no inhibition of viability in HaCaT (Figure 1B, left and right).

### 3.2. KD87 Induces Biochemical Features of Apoptosis in A375 and A431 Cells

**Figure 2:**
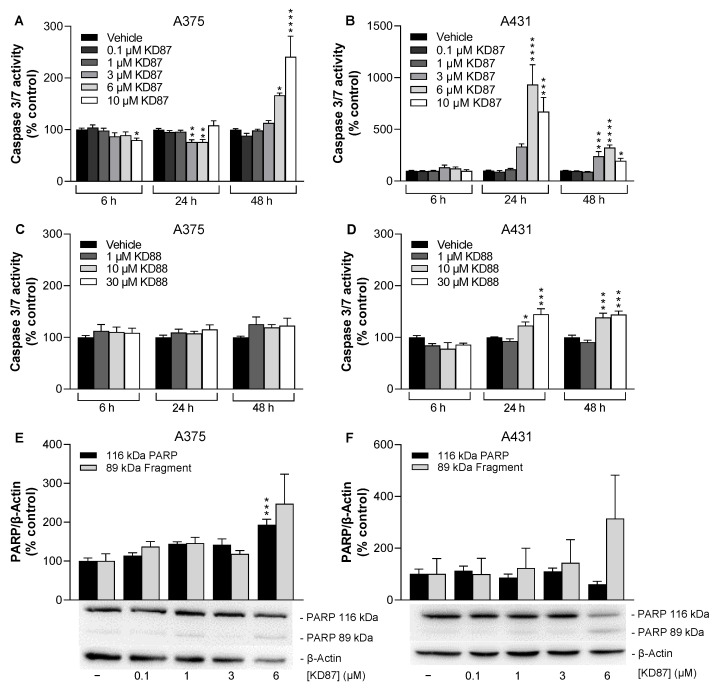
Effect of KD87 and KD88 on features of apoptosis in A375 and A431 cells. Cells were incubated with KD87 or KD88 at the indicated concentrations for 48 h (**E**), 24 h (**F**) or for the indicated incubation periods (**A**–**D**). The values given in the diagrams are based on caspase-3/-7 activity assays (**A**–**D**) or the densitometric analysis of Western blots (**E**,**F**). PARP levels (**E**,**F**) were normalised to β-actin. All percentage values shown refer to the respective vehicle control, which was set to 100%. The blots shown are representative. The data are mean values ± SEM of *n* = 9 per group from 3 independent experiments (**A**–**C**), *n* = 5–6 (**D**) per group from 2 independent experiments or *n* = 3 per group from 3 independent experiments (**E**,**F**). * *p* ≤ 0.05, ** *p* ≤ 0.01, *** *p* ≤ 0.001, **** *p* ≤ 0.0001 vs. corresponding vehicle control; one-way ANOVA with Dunnett’s post hoc test.

The analysis of effector caspases-3 and -7 revealed the increased activation of the latter in KD87-treated A375 (Figure 2A) and A431 cells (Figure 2B), with a comparatively earlier and stronger induction in A431 cells after 24 h of incubation. Significant caspase activity was also observed in KD88-treated A431 (Figure 2D), but not in A375 cells (Figure 2C), although at a level greatly below the effects recorded after KD87 treatment. The significant activation of caspase-3/-7 activity in HaCaT was only registered in the presence of the highest tested KD87 concentration of 10 µM (Supplementary Figure S1A). In contrast, the KD87 concentration of 6 µM, which was used for subsequent studies in skin cancer cells, did not elicit significant upregulation at any of the time points tested. As expected, KD88 showed no proapoptotic effect on HaCaT (Supplementary Figure S1B).

To further analyse a downstream target of effector caspases, poly(ADP-ribose) polymerase (PARP) cleavage was investigated in KD87-treated cells. Thereby, an increase in the cleaved 89 kDa PARP fragment at the times of maximal caspase-3 and -7 induction could be detected in both cell lines for 6 µM KD87, albeit with comparatively high variances (Figure 2E,F). The corresponding effect was more pronounced in A431 cells.

### 3.3. KD87 Inhibits Colony Formation of A375 and A431 Cells

**Figure 3:**
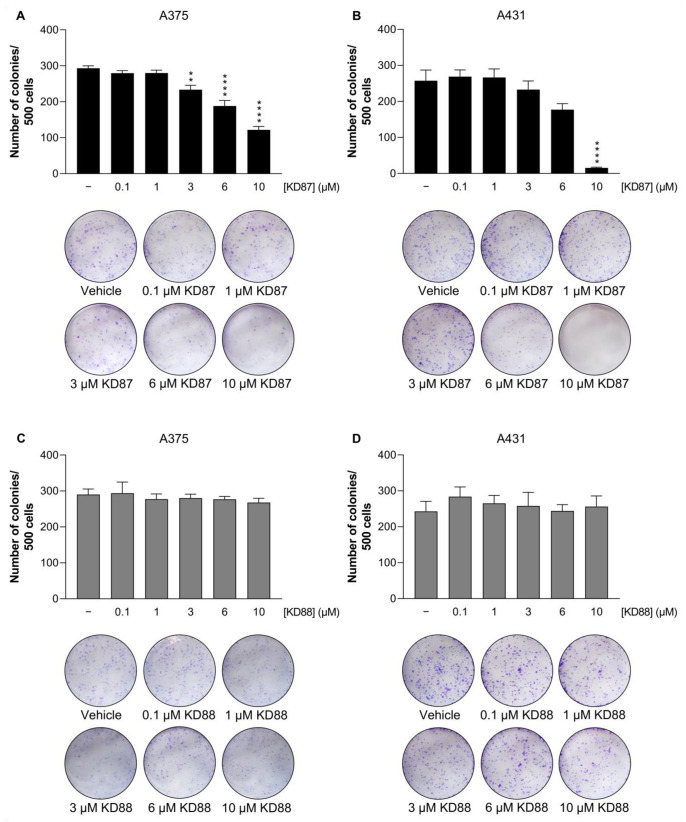
Impact of KD87 and KD88 on colony-forming properties of A375 and A431 cells. For this purpose, 500 cells were seeded on Petri dishes and treated with increasing concentrations of KD87 (**A**,**B**) and KD88 (**C**,**D**) or with vehicle in serum-containing medium for 7 days. Afterwards, the cells were fixed and stained with crystal violet. Colonies were counted as the total number per Petri dish. Values are mean values ± SEM of *n* = 4 independent experiments. ** *p* ≤ 0.01, **** *p* ≤ 0.0001 vs. corresponding vehicle control; one-way ANOVA with Dunnett’s post hoc test.

To determine the influence of KD87 and KD88 on the clonogenic properties of skin cancer cells independent of the specific cell-death mechanism, colony formation experiments were performed next. Since its initial description [28], this assay has been considered the gold standard for determining the effects of ionising radiation therapy on in vitro cellular systems, but is also used to determine the efficacy of cytotoxic agents [29]. The aim was to determine the influence of indirubin derivatives on the proportion of cells that have the ability to form a colony from a single cell and, thus, lead to tumour recurrence. In these experiments, KD87 showed an inhibitory effect on the colony-forming properties of A375 and A431 cells at concentrations between 3 and 10 µM, as shown by the bar graphs in Figure 3A,B and the representative images below. When tested at the highest concentration of 10 µM, the effect of KD87 was clearly more pronounced in A431 cells (Figure 3B) than in A375 cells (Figure 3A). In contrast, no inhibitory effects could be detected in cells treated with KD88 (Figure 3C,D).

### 3.4. KD87 Does Not Inhibit Invasion and Migration of A375 Cells

A major obstacle to the successful treatment of melanoma is its strong invasive potential, which often leads to metastasis (for review see [30]). For this reason, further experiments were conducted with the A375 melanoma cell line to investigate possible effects of KD87 on cancer cell invasion. However, KD87 showed no effect on the invasion of A375 cells through Matrigel-coated inserts and also left the migration of cells through uncoated inserts unaffected (Supplementary Figure S2). At the high cell densities used in the invasion and migration assays, cytotoxicity of KD87 could no longer be detected in a comparable experimental setting (i.e., 250,000 cells per well of a 48-well plate) (Supplementary Figure S2), which is consistent with previous findings by our group on the loss of the viability-reducing effect of cannabinoids when using high cell numbers [27,31,32,33,34].

### 3.5. KD87 Causes Inhibition of Mitochondrial Respiration in A375 and A431 Cells

**Figure 4:**
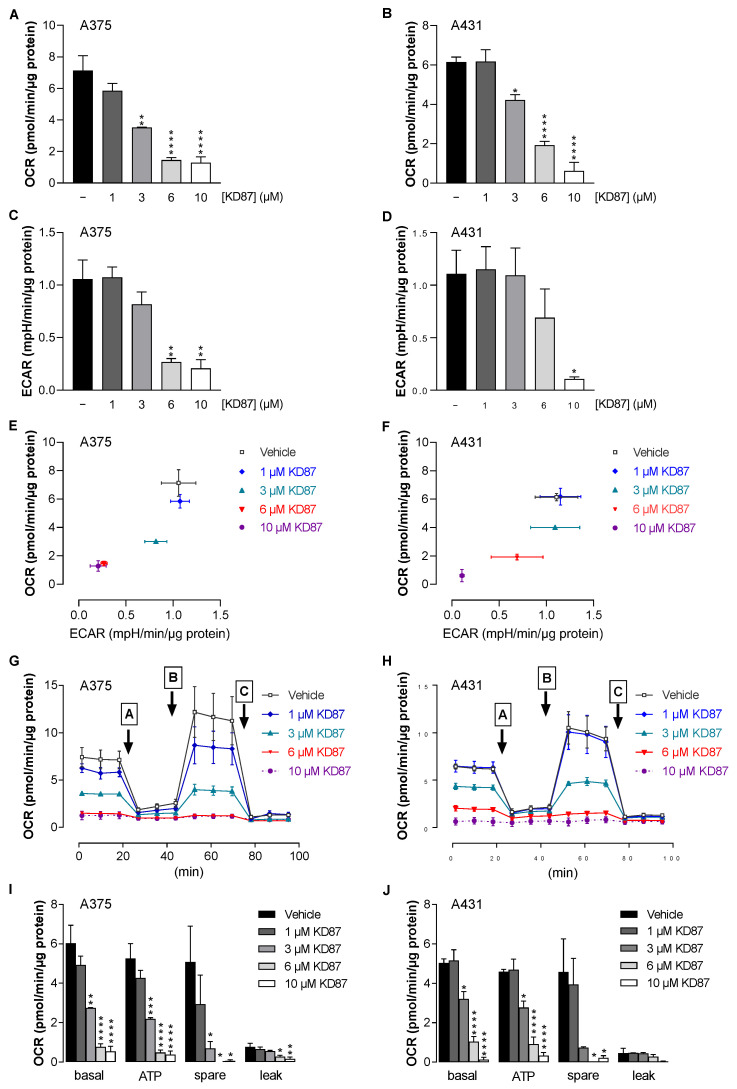
Influence of KD87 on oxygen consumption rate (OCR) and extracellular acidification rate (ECAR) of human melanoma and squamous skin carcinoma cells. A375 and A431 cells were incubated with KD87 at indicated concentrations or the vehicle control for 24 h. Thereafter, OCR (**A**,**B**) and ECAR (**C**,**D**) were determined using the Seahorse XFe24 Analyser. The effects of treatment groups on the OCR and ECAR are summarised in the energy maps in panels (**E**,**F**). Subsequently, a mitochondrial stress test was performed. For this purpose, oligomycin (port A), FCCP (port B) and antimycin A/rotenone (port C) loaded into the respective port of sensor cartridges were released into the wells at the indicated times. In this context, panels (**G**,**H**) show the time courses of the OCR in both cell lines treated with KD87. Panels (**I**,**J**) present calculations of the basal respiration (basal), ATP-bound respiration (ATP), reserve respiratory capacity (spare) and proton leak. The data represent mean values ± SEM of *n* = 3 per group from 3 independent experiments (**A**–**J**). * *p* ≤ 0.05, ** *p* ≤ 0.01, *** *p* ≤ 0.001, **** *p* ≤ 0.0001 vs. corresponding vehicle control; one-way ANOVA with Dunnett’s post hoc test.

The data from this study, and a more recent study by our group suggesting metabolic defects caused by KD87 [20], raised the question of whether KD87 interferes with energy production in the form of intracellular adenosine triphosphate (ATP), which is essential for tumour development. For this purpose, the oxygen consumption rate (OCR) as an indicator of mitochondrial respiration, and the extracellular acidification rate (ECAR) as an indicator of glycolysis were determined. Measurements performed with the Seahorse analyser XFe24 revealed a concentration-dependent inhibition of OCR (Figure 4A,B) and ECAR (Figure 4C,D) by KD87 in both cell lines, so that cancer cells treated with KD87 cannot maintain their previously energy-rich profile via either metabolic pathway. The concentration-dependent shift of metabolism from the high-energy profile (top right) to the quiescent profile (bottom left) induced by KD87 is shown again for both cell lines using the cell-energy phenotype profile in Figure 4E,F.

To gain a more detailed insight into mitochondrial function, a mitochondrial stress test was performed, which, in addition to basal mitochondrial respiration, also includes the parameters ATP-linked respiration, maximum and reserve capacity and non-mitochondrial respiration. The OCR curves resulting from the time-delayed addition of artificial modulators of oxidative phosphorylation are depicted in Figure 4G,H. According to Figure 4I,J, the incubation of A375 and A431 cells with KD87 decreased the OCR values of basal respiration, ATP production and reserve respiratory capacity in a concentration-dependent manner. The inhibition of the reserve respiratory capacity, which already started at a concentration of 1 µM, clearly showed the strong impairment of cellular respiration by KD87. The proton leak remained relatively constant up to a concentration of 3 µM, but showed decreasing values at higher concentrations of KD87 (Figure 4I,J).

Overall, the data obtained with the Seahorse XFe24 Analyser suggest mitochondrial damage triggered by KD87.

### 3.6. KD87 Confers Structural Mitochondrial Changes in A375 and A431 Cells

**Figure 5:**
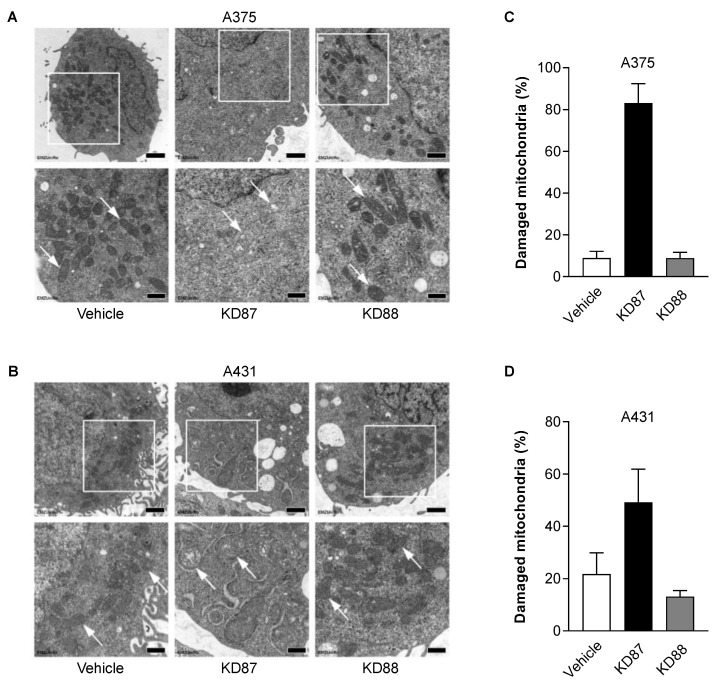
Influence of KD87 and KD88 on the mitochondrial structure of human melanoma and squamous skin carcinoma cells. Representative images from the transmission electron microscopy of A375 (**A**) and A431 cells (**B**) treated for 24 h with vehicle, 6 µM KD87 or 6 µM KD88. The bottom images in (**A**,**B**) are enlarged views of the outlined areas in the upper images of these panels. The structures highlighted with white arrows in the bottom images repredsent intact mitochondria (vehicle, KD88) vs. damaged mitochondria (KD87). Scale bars in (**A**,**B**) are 1 µm (**upper** images) or 500 nm (**lower** images). The number of damaged mitochondria is presented as a percentage of the total number of mitochondria in the respective images examined (**C**,**D**). Each analysed image is from a different cell. The data are given as mean values ± SEM of *n* = 11–15 (**C**) or *n* = 12–14 (**D**) images.

In order to obtain a visual impression of the mitochondria-damaging effect of KD87 proven in the Seahorse analyses, electron microscopic examinations were carried out in a further step (Figure 5). In the electron micrographs, compact mitochondria with electron-dense, osmiophilic staining were clearly visible in the vehicle-treated controls of both cell lines (Figure 5A,B, white arrows). In contrast, the mitochondria in KD87-exposed cells were frequently characterised by a loss of electron-dense, osmiophilic staining, therefore appearing brighter, with significant swelling and defective cristae and membranes (Figure 5A,B, white arrows). The percentage of damaged mitochondria relative to the total number of mitochondria in the analysed images is shown in Figure 5C,D for cells treated with vehicle, KD87 or KD88, using the mitochondrial membrane integrity, degree of swelling and cristae structure integrity as parameters for quantification. Here, a clear increase in damaged mitochondria was observed in both cell lines after treatment with KD87 compared to the vehicle control. Cells treated with KD88 largely showed no morphological changes in mitochondrial structures compared to vehicle (Figure 5A–D).

### 3.7. KD87 Induces HO-1 Expression, Whose Inhibition Leads to a Partial Restoration of the Decreased Metabolic Activity of A375 and A431 Cells Caused by KD87

In the context of the effect of KD87, further studies focused on a possible role of the enzyme HO-1, which exerts an influence on mitochondrial biogenesis [35,36,37,38,39,40], induces mitophagy [41] and, according to recent studies, may also have a proapoptotic effect on cancer cells [42,43,44].

**Figure 6:**
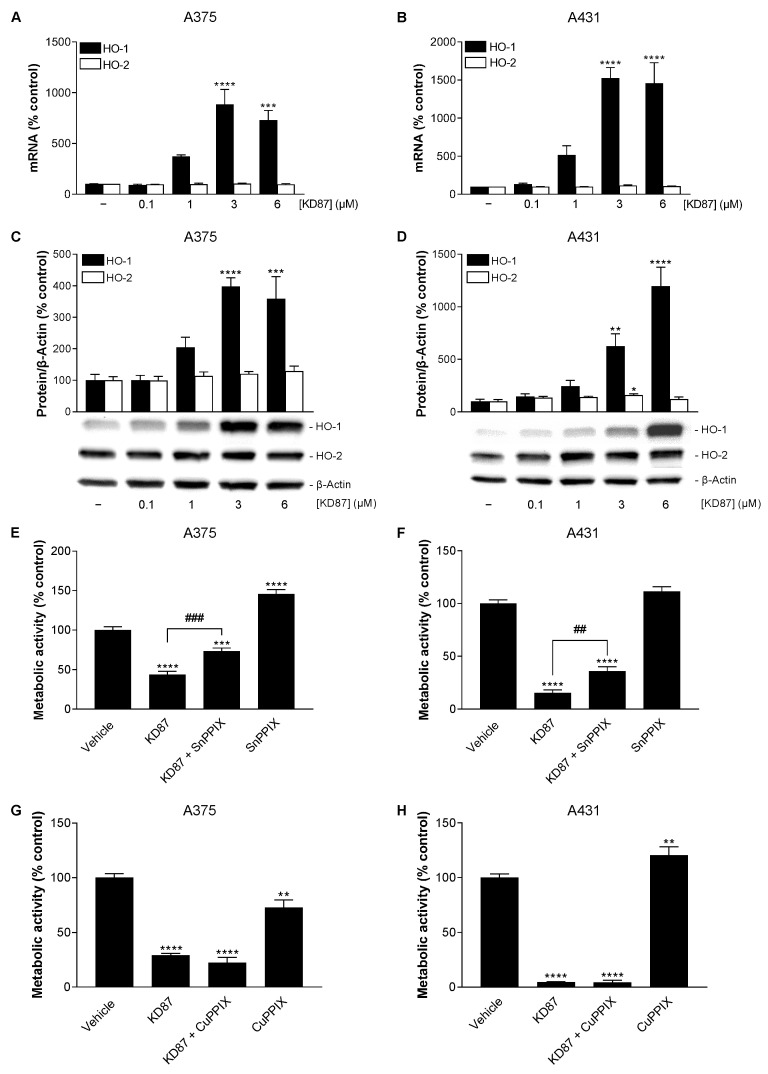
The effect of KD87 on the expression of HO-1 and HO-2 at the mRNA (**A**,**B**) and protein (**C**,**D**) level and influence of the HO-1 inhibitor SnPPIX (**E**,**F**) or its non-HO-1-inhibiting analogue CuPPIX (**G**,**H**) on the KD87-mediated decrease in metabolic activity of A375 and A431 cells. Cells were incubated with KD87 at the indicated concentrations (**A**–**D**) concentrations or at 6 µM (**E**–**H**) for 24 h (**A**–**D**,**F**,**H**) or 48 h (**E**,**G**). In the inhibitor experiments (**E**–**H**), cells were pre-treated with SnPPIX (25 µM), CuPPIX (25 µM) or vehicle for 1 h, followed by a 24 h or 48 h co-incubation with KD87 or vehicle. The values given in the bar charts are based on quantitative RT-PCR (**A**,**B**), the densitometric analyses of blots (**C**,**D**) or WST-1 assay experiments (**E**–**H**). HO mRNA levels (**A**,**B**) were normalised to PPIA mRNA levels and HO protein levels (**C**,**D**) to β-actin. All percentage values shown refer to the respective vehicle control, which was set to 100%. The blots shown are representative. The mRNA and protein data are mean values ± SEM of *n* = 4 (**A**,**B**), *n* = 8 (**C** (HO-1)), *n* = 6 (**C** (HO-2)), *n* = 6 (**D** (HO-1)) or *n* = 5 (**D** (HO-2)) independent experiments. Metabolic activity data are mean values ± SEM of *n* = 20 (**E**) or *n* = 18–20 (**F**) per group from 5 independent experiments each, and *n* = 8–9 (**G**) or *n* = 9 (**H**) per group from 3 independent experiments each. * *p* ≤ 0.05, ** *p* ≤ 0.01, *** *p* ≤ 0.001, **** *p* ≤ 0.0001 vs. corresponding vehicle control; ## *p* ≤ 0.01, ### *p* ≤ 0.001 vs. corresponding KD87-treated group; one-way ANOVA with Dunnett’s (**A**–**D**) or Bonferroni’s (**E**–**H**) post hoc test.

To this end, the expression level of this enzyme in the presence of KD87 was first analysed at the mRNA and protein level using RT-PCR and Western blot. Thereby, a corresponding concentration-dependent HO-1 induction caused by KD87 could be detected in A375 and A431 cells both at the mRNA (Figure 6A,B) and protein level (Figure 6C,D), whereas the parallel-measured HO-2 isoenzyme was not subject to a corresponding regulation. At the functional level, the inhibition of HO-1 by the HO-1 activity inhibitor SnPPIX resulted in a partial but significant inhibition of the KD87-mediated decrease in metabolic activity of A375 and A431 cells (Figure 6E,F), whereas the non-HO-1-inhibiting negative control CuPPIX was virtually inactive in this regard (Figure 6G,H).

### 3.8. N-Acetyl-L-Cysteine Attenuates the Metabolic-Activity-Reducing and HO-1-Upregulating Effect of KD87 on A375 and A431 Cells

**Figure 7:**
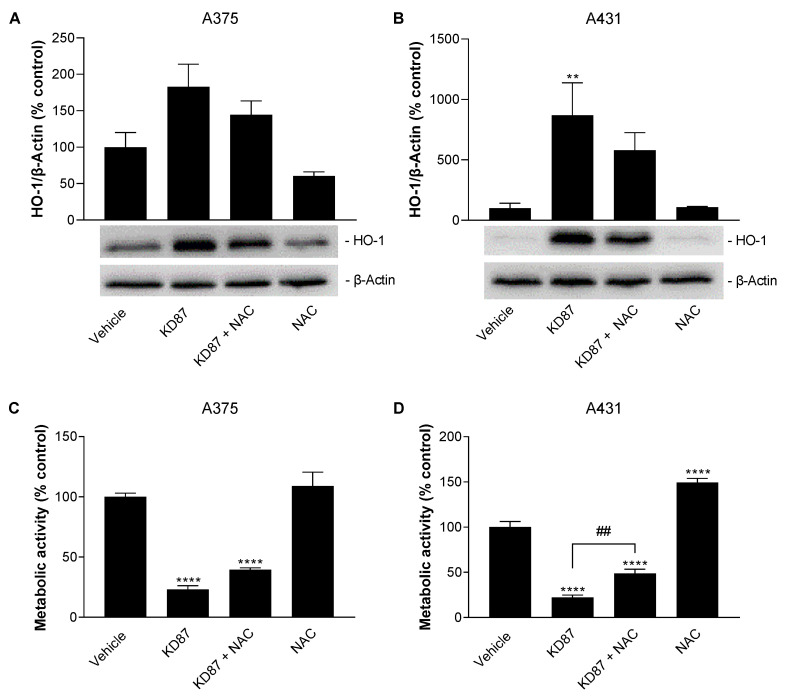
Influence of *N*-acetyl-L-cysteine (NAC) on HO-1-upregulating (**A**,**B**) and metabolic activity-reducing effects (**C**,**D**) of KD87 in A375 and A431 cells. Cells were pretreated with NAC (1 mM) or vehicle for 1 h followed by a 24 h (**A**,**B**,**D**) or 48 h (**C**) co-incubation with KD87 (6 µM) or vehicle. The values given in the bar charts are based on the densitometric analyses of blots (**A**,**B**) or WST-1 tests (**C**,**D**). HO-1 protein levels (**A**,**B**) were normalised to β-actin. All percentage values shown refer to the respective vehicle control, which was set to 100%. The blots shown are representative. The data are mean values ± SEM of *n* = 5 (**A**,**B**) per group of 5 independent experiments, or *n* = 9 (**C**,**D**) per group of 3 independent experiments. ** *p* ≤ 0.01, **** *p* ≤ 0.0001 vs. corresponding vehicle control; ## *p* ≤ 0.01 vs. corresponding KD87-treated group; one-way ANOVA with Bonferroni’s post hoc test.

Considering the dependence of HO-1 expression on reactive oxygen species (ROS), shown in different types of cells [45,46,47,48], the impact of the ROS scavenger *N*-acetyl-L-cysteine (NAC) on the KD87-induced HO-1 protein formation was investigated next. This resulted in a 47% (A375) and 38% (A431) reduction in KD87-induced protein levels (Figure 7A,B), although these effects did not reach statistical significance. Also at the functional level, a partial inhibition of the KD87-induced reduction in metabolic activity by 21% (A375) and 34% (A431) was demonstrated upon co-incubation with NAC (Figure 7C,D), with a significant effect in A431 cells.

### 3.9. Inhibition of HO-1 Causes a Partial but Not Significant Inhibition of KD87-Induced Release of Mitochondrial Cytochrome c in A375 and A431 Cells

HO-1 is an enzyme that can exert different functions depending on its localisation. To gain further insight into the role of mitochondrial HO-1, its putative translocation to mitochondria upon the exposure of cells to KD87 was determined, as was its influence on the translocation of cytochrome c from mitochondria to the cytosol. For this purpose, both cell lines were treated again with KD87, the HO-1 inhibitor SnPPIX or both substances together, with the subsequent determination of the corresponding mitochondrial proteins following fractionation of the cell organelles.

**Figure 8:**
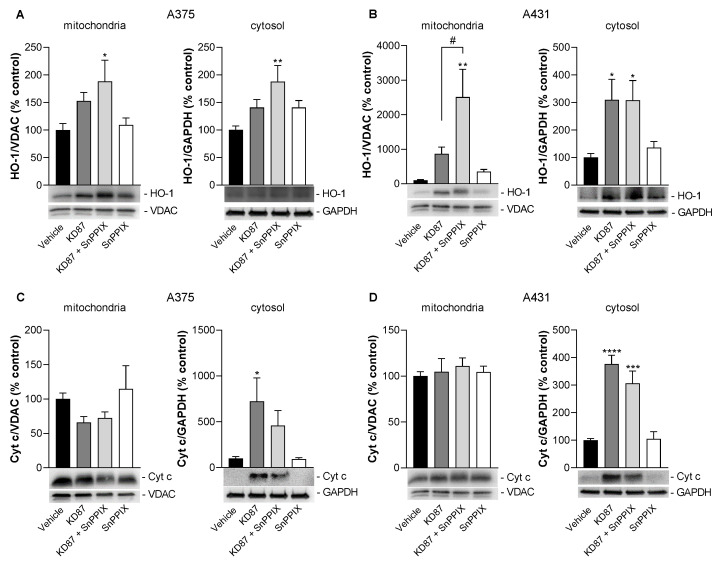
The effect of KD87 alone or in the presence of the HO-1 inhibitor SnPPIX on the protein concentrations of HO-1 (**A**,**B**) and on the release of mitochondrial cytochrome c (Cyt c) into the cytosol (**C**,**D**) of A375 and A431 cells. The cells were pretreated for 1 h with SnPPIX (25 µM) or vehicle followed by a 24 h co-incubation with KD87 (6 µM) or vehicle. Subsequently, the corresponding proteins in the mitochondrial and cytosolic fractions were determined using Western blot. The values given in the bar charts are based on the densitometric analyses of blots. Mitochondrial HO-1 and Cyt c levels were normalised to VDAC, whereas the same proteins in the cytosol were normalised to GAPDH. The blots shown are representative. In (**A**,**C**), the same GAPDH blots, and in (**B**,**D**), the same VDAC blots as well as the same GAPDH blots, are shown, since the proteins analysed in these Western blots were resolved on the same gel. The data are mean values ± SEM of *n* = 7 ((**A**,**B**), right graph; (**C**), left graph) or *n* = 8 ((**B**), left graph; (**C**), right graph; (**D**)) of the group from 7 or 8 independent experiments. All percentage values shown refer to the respective vehicle control, which was set to 100%. * *p* ≤ 0.05, ** *p* ≤ 0.01, *** *p* ≤ 0.001, **** *p* ≤ 0.0001 vs. corresponding vehicle control; # *p* ≤ 0.05 vs. corresponding KD87-treated group; one-way ANOVA with Bonferroni’s post hoc test.

As shown in Figure 8A,B, a profound upregulation of HO-1 by 6 µM KD87 was detected in mitochondrial fractions, which was enhanced in the concomitant presence of SnPPIX. SnPPIX alone similarly caused an increase in HO-1 formation when incubated alone, which was registered in either the cytosolic fraction (A375) or cytosolic and mitochondrial fractions (A431). However, the induction of HO-1 transcription by SnPPIX is well described in the literature, where the efficiency of the inhibitor appears to be sufficient for blocking both the preformed HO-1 enzyme and the HO-1 enzyme synthesised de novo under the aforementioned circumstances [49,50].

In agreement with the microscopically detected mitochondrial damage, a release of the mitochondrial cytochrome c into the cytosol of A375 and A431 cells was also caused by KD87 (Figure 8C,D), which, furthermore, may explain the activation of effector caspases previously shown (Figure 2A,B) as a marker of mitochondrial apoptosis. In our case, SnPPIX attenuated the cytosolic increase in cytochrome c caused by KD87 by 42% (Figure 8C) and 25% (Figure 8D), respectively, although neither inhibition was statistically significant. However, because the release of cytochrome c induced by KD87 is indicative of mitochondrial dysfunction, the partial inhibitions by SnPPIX shown here agree well with the partial restoration of metabolic activity by SnPPIX in cells treated with KD87 shown before (Figure 6E,F).

### 3.10. KD87 Leads to the Downregulation of Certain Subunits of the Respiratory Chain Complex in A375 and A431 Cells

**Figure 9:**
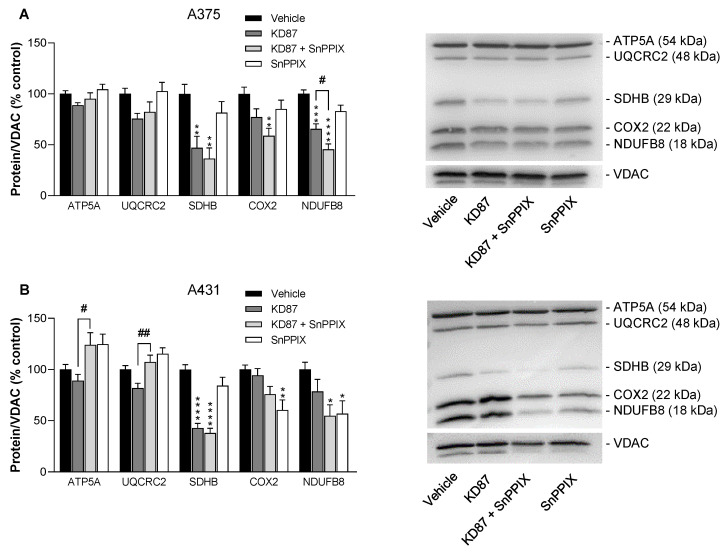
The effect of KD87 alone or in the presence of the HO-1 inhibitor SnPPIX on the concentrations of subunits of mitochondrial respiratory chain complexes of A375 (**A**) and A431 cells (**B**). The cells were treated for 24 h with the indicated concentrations of KD87. Subsequently, the corresponding proteins in the mitochondrial fractions were determined using Western blot. The values given in the bar charts are based on the densitometric analyses of blots. Mitochondrial proteins were normalised to VDAC. All percentage values shown refer to the respective vehicle control, which was set to 100%. The blots shown are representative. The data are mean values ± SEM of *n* = 7 (**A**) or *n* = 8 (**B**) per group from 7 or 8 independent experiments. * *p* ≤ 0.05, ** *p* ≤ 0.01, *** *p* ≤ 0.001, **** *p* ≤ 0.0001 vs. corresponding vehicle control; # *p* ≤ 0.05, ## *p* ≤ 0.01 vs. corresponding KD87-treated group; one-way ANOVA with Bonferroni’s post hoc test.

To further characterise the reduction in OCR, the effect of KD87 alone and in combination with the HO-1 inhibitor SnPPIX on the levels of specific subunits of the respiratory chain complexes was investigated on protein extracts from isolated mitochondria using an OxPhos antibody set. This revealed a significant reduction in succinate dehydrogenase complex subunit B (SDHB, subunit of complex II) in A375 and A431 cells (Figure 9A,B) and a significant decrease in NADH:ubiquinone oxidoreductase subunit B8 (NDUFB8, subunit of complex I) in A375 cells (Figure 9A). However, SnPPIX did not restore the subunits significantly reduced by KD87 in A375 and A431 (Figure 9A,B). In addition, KD87 caused larger 20% reductions, albeit non-significant, in ubiquinol-cytochrome c reductase core protein 2 (UQCRC2, subunit of complex III) and cytochrome c oxidase subunit 2 (COX2, subunit of complex IV) in A375 cells, as well as in NADH:ubiquinone oxidoreductase subunit B8 (NDUFB8, subunit of complex I) in A431 cells (Figure 9A,B). In both cell lines, mitochondrial ATP synthase subunit 5A (ATP5A) was only reduced by about 10% non-significantly by KD87.

## 4. Discussion

There is a need to develop new therapeutic options for the treatment of skin cancer. In the study presented here, KD87, a member of the thia-analogue indirubin *N*-glycoside class, is characterised as a potent cytotoxic agent for skin cancer cells, causing various impairments in mitochondrial function and integrity. The established cell lines A375 and A431 were used to represent melanoma and cutaneous SCC, respectively, as an approach to evaluate the compound’s effectiveness against different skin cancers. The efficiency of KD87 in reducing cell viability by inducing mitochondrial dysfunction was comparable in both cell lines, e.g., in terms of the morphologically detected increase in defective mitochondria, concentration-dependent reductions in basal and ATP-linked OCR and the reserve capacity of oxidative respiration, the release of mitochondrial cytochrome c into the cytosol, and a decrease in SDHB, a subunit of complex II.

In our hands, KD87 was shown to increase the activity of executioner caspases-3 and -7 and PARP cleavage, accompanied by the release of mitochondrial cytochrome c into the cytosol, thereby suggesting a mitochondrial pathway of apoptosis. Apoptosis mechanisms for various glycoside-linked indirubin 4-methoxyacetophenone derivatives have also been previously examined in melanoma cells [19,24] and T-cell lymphoma cells [23]. Here, the work with melanoma cells focused on the sensitising effect of corresponding indirubin derivatives on death ligand-induced apoptosis.

KD87 even showed an inhibitory effect in colony formation assays, suggesting that the compound significantly reduces the clonal proliferation ability of skin cancer cells. On the other hand, the invasion and migration of A375 cells remained unaffected by KD87. The lack of effect of KD87 on the invasiveness of A375 cells in the present study is in contrast to results of other authors, who reported an anti-invasive effect of I3M on pancreatic ductal adenocarcinoma cells in vitro and in vivo [51] and on oral cancer cells [52], as well as of 6-bromo-indirubin-3’-oxime on the invasiveness of colorectal cancer cells [53] and metastasis in a murine breast cancer model [54]. Interestingly, in the latter study, 6-bromo-indirubin-3’-oxime was found to inhibit the migration of urinary bladder carcinoma, hepatocarcinoma, triple-negative breast cancer, and murine mammary carcinoma cell lines. This discrepancy suggests that KD87 has a mechanism of action that circumvents these effects on cancer cell migration and invasion.

The present study has also shown that the glycosylation of the compound is important for the function of the molecule, making it an ideal site for chemical modification to further enhance or control its activity. In this respect, the inability of KD88, a non-glycosylated thia-analogous indirubin, to induce cell death is in agreement with a previous work in which, using the sulforhodamine B cytotoxicity assay [55], the IC_50_ values for unsubstituted indirubin were >30 µM (A549 lung cancer cells, HT1080 fibrosarcoma cells) or >100 µM (SNU-638 gastric cancer cells, Col2 colon cancer cells, MCF-7 breast cancer cells and HL60 leukaemia cells). Our data now also prove the importance of glycosylation of the indirubin structure for triggering decreased colony formation and mitochondrial defects in melanoma and SCC cells. At present, the exact function of glycosylation is not yet clear, but it is evident that it increases water solubility. Indeed, the development of more water-soluble indirubin derivatives is also expected to have clinical benefits. Thus, the severe gastrointestinal side effects of indirubin are often mentioned in the context of its poor water solubility [6,7,10,56].

Although preceding studies have already pointed to metabolic defects on the whole-cell level in response to KD87 treatment [20,21], the detailed examination of mitochondrial morphology in electron micrographs presented here, combined with an in-depth analysis of mitochondrial respiration using the Seahorse analyser, now suggests that KD87 severely impairs mitochondrial function. It was clear from mitochondrial morphology and energetic deficiencies, as well as the presence of cytochrome c in the cytoplasm, that mitochondrial damage is a major promoter of cell death in KD87-treated cells. Thus, it was shown that the cells were unable to compensate the energy depletion due to loss of oxidative phosphorylation (OCR) by the upregulation of glycolysis (ECAR). In addition, damage to mitochondria by KD87 was demonstrated to lead to the significant downregulation of SDHB, a subunit of respiratory chain complex protein II, in A375 and A431 cells and of NDUFB8, a subunit of complex I, in A375 cells. A mitochondrial-associated effect of indirubins has also been confirmed by the studies of other authors. Thus, a mitochondrial-mediated effect with the inhibition or loss of the mitochondrial membrane potential could be demonstrated for I3M in prostate cancer and human embryonic kidney (HEK) cells [25], as well as for its derivative 5-diphenylacetamido-indirubin-3’-oxime (LDD398) in a myeloid leukaemia cell line [26].

Another result of the present work is the concentration-dependent increase in HO-1 mRNA and protein expression by KD87 in A375 and A431 cells. Thereby, in addition to the upregulation found in whole cell lysates, an increase in HO-1 was also observed in mitochondrial fractions. Noteworthy, the enzymatic activity of HO-1 is retained even after translocation into the mitochondria, while the truncation necessary for migration into the nucleus leads to an enzymatically inactive, but still gene-modulatory effective HO-1 (for review see [57,58]). The translocation of HO-1 to mitochondria has been described in response to very severe cellular stress conditions such as oxidative stress [39], hypoxia [39], lipopolysaccharide [35] and cigarette smoke [59]. In one of these studies, mitochondria-targeted HO-1 was linked to oxidative stress and mitochondrial dysfunction under normoxic conditions, in contrast to the protective effect of endoplasmic reticulum-associated HO-1 [39]. Consistent with this, increased mitochondrial HO-1 expression was also associated with higher ROS production and the increased mitochondrial recruitment of autophagy markers LC-3 and Drp-1, suggesting increased mitophagy or autophagy [39]. Yet another work suggested a potential role of mitochondrial HO-1 in defence against further mitochondria-mediated cell death [59]. However, in our study, oxidative stress could only be detected to a limited extent through the partial inhibition of the KD87-induced reduction in metabolic activity by the ROS scavenger NAC. On the other hand, ROS also appear to have a stimulatory effect on HO-1 expression, as demonstrated by the inhibition of HO-1 formation by NAC, e.g., in renal tubule epithelial [45], umbilical vein endothelial [48], smooth muscle [47] and HepG2 hepatoma cells [46]. The fact that HO-1 expression induced by KD87 was at least partially inhibited by NAC in both A375 and A431 cells suggests that oxidative signalling represents one, but not the only, underlying mechanism. Incidentally, other anticancer drugs also appear to disrupt the mitochondrial function of tumour cells as part of their action via the translocation of HO-1 into mitochondria, as has been shown for the isoflavone derivative ME-344 [60] and the nuclear factor (NF)-κB inhibitor BAY114085 [61].

In the present study, a possible adverse effect of HO-1 is supported by the finding that the inhibition of its enzymatic activity by SnPPIX resulted in a partial reversal of two detrimental effects by KD87, namely the decrease in metabolic activity and the increase in the release of cytochrome c from the mitochondrion into the cytosol. Interestingly, there has recently been an accumulation of findings confirming [42,43,44] or suggesting [62,63,64] a proapoptotic role of HO-1 on tumour cells. On the other hand, HO-1 can also play a specific role in cancer progression (for reviews see [65,66]), for example, in promoting tumour angiogenesis in pancreatic cancer [67]. The effect of the enzyme, thus, varies between different cancer types. Furthermore, the subcellular localisation of HO-1 may explain the different effects attributed to the protein in different tumour types, with the nuclear localisation of HO-1 being involved in processes leading to cancer progression (for review see [58]). Still, further studies need to uncover the exact role of KD87-induced HO-1 in regulating skin-cancer-cell viability, while also investigating other mitochondrial factors.

In contrast to mitochondrial cytochrome c release, the HO-1 inhibitor SnPPIX showed no measurable reversal effect on the KD87-induced significant reduction in the complex II protein SDHB in A375 and A431 cells and the complex I protein NDUFB8 in A375 cells. So far, both stimulatory [36,38,40] and inhibitory effects [35,37,39] of HO-1 on mitochondrial biogenesis have been described in the literature. In the latter studies, the induction of mitochondrial HO-1 was associated with a decrease in cytochrome c oxidase (COX) activity [39] and the expression of heme-containing complex III [39] and heme-containing COX subunits I [35,37] and II [37], probably due to heme clearance by HO-1, with minimal effects on the non-heme-containing proteins citrate synthase and VDAC [37]. On the other hand, HO-1 or its product CO is attributed an activating role in mitochondrial biogenesis (for review see [68]) via increasing mitochondrial ROS production, peroxisome receptor γ coactivator 1α (PGC-1α), nuclear respiratory factor-1 (NRF-1) and mitochondrial transcription factor A (TFAM) [38,40].

Our series of experiments also included experiments with human HaCaT keratinocytes, which should actually exclude the effects of KD87 on non-malignant skin cells. Consistent with this expectation, no significant reduction in viability by KD87 up to a concentration of 6 µM was observed in these cells after 24 h of incubation, in contrast to A375 and A431 cells. Moderately lower toxicity compared to A431 cells was observed after a 48 h incubation, although HaCaT cells were also significantly affected here by treatment with KD87 at concentrations of 6 and 10 µM. The toxicity of potential skin cancer drugs on HaCaT cells was already reported in the literature [69,70], but hardly discussed. For example, Olivan-Viguera et al. [71] studied TRPV4 agonists initially as potential drugs in melanoma, but then found that they induced apoptosis and necrosis equally in A375 and HaCaT. Their effect on keratinocytes was eventually discussed as an option to prevent hyperkeratosis in autoimmune diseases such as psoriasis [71]. Interestingly, indirubin also proved to be an inhibitor of the proliferation of HaCaT cells [72,73] so that an anti-psoriasis potential can also be considered for this group of substances. In the case of A375, it has recently been pointed out that for this cell type, non-malignant primary melanocytes must in principle be used as controls [74], which is not performed in most studies, including in the present one. Therefore, and considering the demonstrated delayed effect of KD87 on HaCaT viability, a definitive conclusion on the selectivity of the effect of KD87 on skin cancer cells cannot be given here.

Further studies should also include other skin cell lines that show resistance to chemotherapeutic agents. Accordingly, indirubin and its derivatives are currently being discussed to overcome imatinib resistance in CML [75]. Other published data have shown that an indigoid derivative can overcome chemoresistance in leukaemia cells [76] and that I3M sensitises multiple myeloma cells to bortezomib-induced apoptosis and overcomes bortezomib resistance via proteasome inhibition [77].

**Figure 10:**
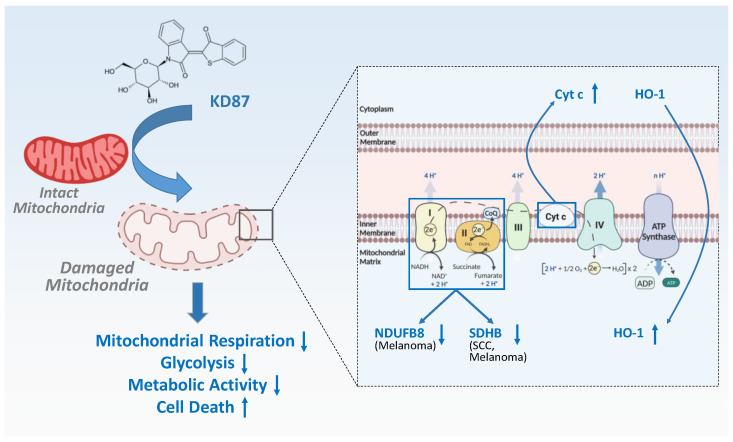
Proposed mechanism of KD87-induced cell death of melanoma and SCC cells of the skin. The glycosylated indirubin derivative KD87 induces mitochondrial damage in melanoma and SCC cells, which is quantitatively measurable by the loss of OCR (indicator of mitochondrial respiration) and ECAR (indicator of glycolysis) and is visible as mitochondrial swelling and the loss of the mitochondrial cristae structure. Mitochondrial damage is also accompanied by an increase in cytochrome c in the cytosol. Major respiratory chain proteins downregulated by KD87 include NDUFB8 and SDHB in melanoma cells, and SDHB in SCC cells. In addition, KD87 causes an increase in HO-1 in the cells, which is also measurable in their mitochondria. The partial reversal of the KD87-induced loss of metabolic activity of the cells by the HO-1 activity inhibitor SnPPIX suggests that this regulation appears to be involved in the cytotoxic effect of KD87. Finally, KD87 showed inhibition of the in vitro colony formation of melanoma and SCC cells, leading to the general hypothesis that KD87 may have a long-term inhibitory effect on tumour recurrence in addition to potential tumour-regressive properties. Abbreviations and arrows: Cyt c, cytochrome c; HO-1, heme oxygenase-1; NDUFB8, NADH:ubiquinone oxidoreductase subunit B8; SCC, squamous cell carcinoma; SDHB, succinate dehydrogenase complex subunit B; ↑ (vertical thin blue), upregulation/increase upon KD87 treatment; ↓ (vertical thin blue), downregulation/decrease upon KD87 treatment. The curved blue arrows represent a translocation of the respective protein. (Created with BioRender.com).

Overall, the data presented here show that the indirubin derivative KD87 triggers a cellular cascade, leading to extensive mitochondrial damage, energy deficit and ultimately cell death (Figure 10). KD87 resulted in damage to mitochondria with a subsequent release of cytochrome c and a decrease in metabolic activity. Although the molecular mechanism by which KD87 triggers cell death has to be further addressed in future studies, it is clear that the indirubin derivative strongly induces cell death in aggressive skin cancer cells, warranting further investigation as part of ongoing efforts to develop and improve anti-cancer drugs.

## Data Availability

Data are available upon reasonable request from the first author.

## Supplementary Materials

The following supporting information can be downloaded at: https://www.mdpi.com/article/10.3390/cells12192409/s1, Figure S1: Effect of KD87 (A) and KD88 (B) on caspase-3/-7 activity in HaCaT cells; Figure S2: The effect of KD87 on invasion, migration and viability of A375 cells under conditions of high cell density.
