# Supplementary material for: A Thia-Analogous Indirubin N-Glycoside Disrupts Mitochondrial Function and Causes the Death of Human Melanoma and Cutaneous Squamous Cell Carcinoma Cells
Source: Cells. 2023 Oct 5;12(19):2409. doi: 10.3390/cells12192409 (PMC10572502; doi:10.3390/cells12192409)
Supplement: Supplementary file 1 [file cells-12-02409-s001.zip › cells-2304751-supplementary.pdf]

cells

Supplementary Material

A Thia-Analogous Indirubin *N*-Glycoside Disrupts Mitochondrial Function and Causes the Death of Human Melanoma and Cutaneous Squamous Cell Carcinoma Cells

Franziska Wendt [1], Felix Wittig [1], Anne Rupprecht [1], Robert Ramer [1], Peter Langer [2], Steffen Emmert [3], Marcus Frank [4,5] and Burkhard Hinz [1,*]

[1] Institute of Pharmacology and Toxicology, Rostock University Medical Centre, 18057 Rostock, Germany; franziska.wendt@med.uni-rostock.de (F.W.); felix.wittig@med.uni-rostock.de (F.W.); rupprechta31@gmail.com (A.R.); robert.ramer@med.uni-rostock.de (R.R.)
[2] Institute of Organic Chemistry, University of Rostock, 18059 Rostock, Germany; peter.langer@uni-rostock.de
[3] Clinic and Policlinic for Dermatology, Rostock University Medical Centre, 18057 Rostock, Germany; steffen.emmert@med.uni-rostock.de
[4] Electron Microscopy Centre, Rostock University Medical Centre, 18057 Rostock, Germany; marcus.frank@med.uni-rostock.de
[5] Department Life, Light and Matter, University of Rostock, 18059 Rostock, Germany
* Correspondence: burkhard.hinz@med.uni-rostock.de

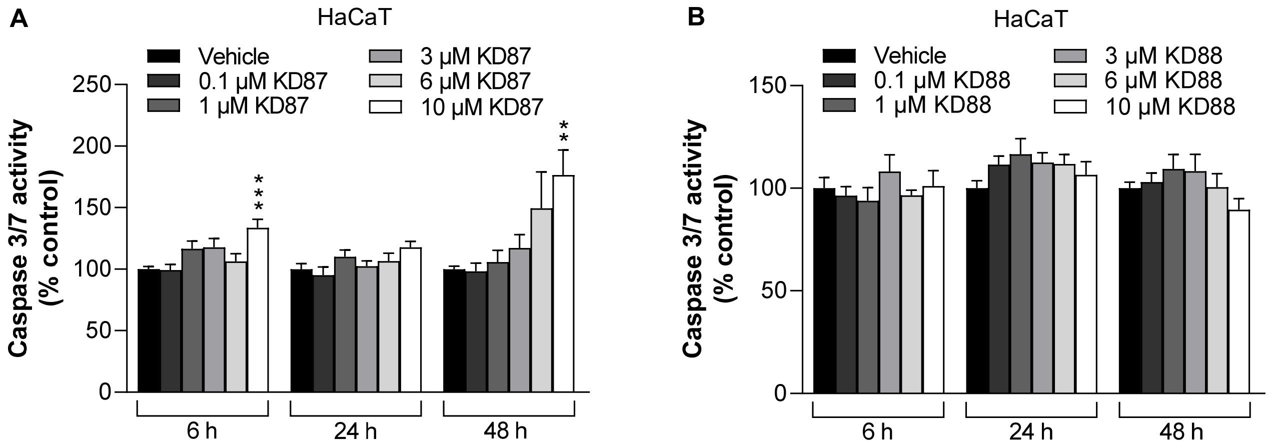

Supplementary Figure S1. Effect of KD87 (**A**) and KD88 (**B**) on caspase-3/-7 activity in HaCaT cells. Cells were incubated with KD87 or KD88 at the indicated concentrations for the indicated incubation periods. The values given in the diagrams are based on caspase-3/-7 activity assays. All percentage values shown refer to the respective vehicle control, which was set to 100%. The data are mean values ± SEM of n = 12 per group from 4 independent experiments. ** $p \leq 0.01$, *** $p \leq 0.001$ vs. corresponding vehicle control; one-way ANOVA with Dunnett's post hoc test.

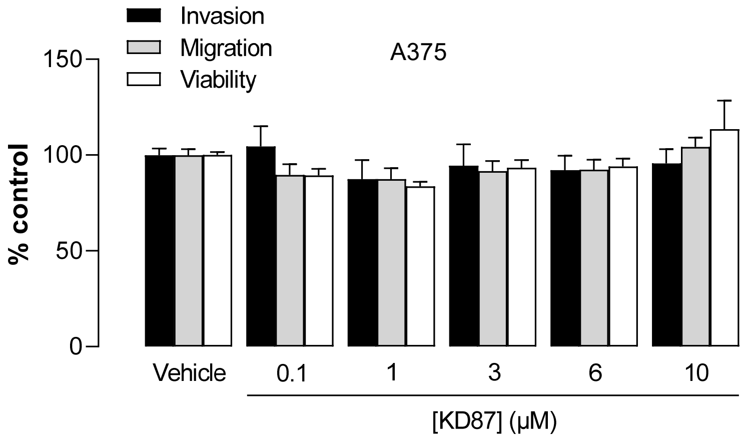

Supplementary Figure S2. The effect of KD87 on invasion, migration and viability of A375 cells under conditions of high cell density. Concentration dependence of KD87 in invasion (black bars) and its effect on cell viability (white bars) and migration through uncoated Boyden chambers (gray bars). A375 cells were incubated with the indicated concentrations of KD87 or vehicle for 72 h. Invasion or migration was measured with modified Boyden chamber assays using Matrigel-coated (invasion) or uncoated transwell inserts. Moreover, viability was quantified using the WST-1 assay to exclude possible toxic effects under comparable conditions of a high cell density (250,000 cells per well of a 48-well plate). All percentage values shown refer to the respective vehicle control, which was set to 100%. The data are mean values ± SEM of n = 8–9 per group from 3 independent experiments, respectively. None of the groups compared to the corresponding vehicle control revealed a significant difference; one-way ANOVA with Dunnett's post hoc test.
